# Effect of Qigong on quality of life: a cross-sectional population-based comparison study in Taiwan

**DOI:** 10.1186/1471-2458-11-546

**Published:** 2011-07-09

**Authors:** Tsung-Jung Ho, David C Christiani, Tso-Chiang Ma, Tsong-Rong Jang, Chih-Hui Lieng, Yi-Chun Yeh, Shinn-Zong Lin, Jaung-Geng Lin, Jim-Shoung Lai, Tzuo-Yun Lan

**Affiliations:** 1 School of Chinese Medicine, China Medical University, No. 91, Hsueh-Shih Road, Taichung 404, Taiwan; 2 Division of Chinese Medicine, Cnina Medical University Beigang Hospital, No. 123, Shin-Der Road, Beigang Town, Yunlin County 651, Taiwan; 3 Environmental & Occupational Medicine & Epidemiology Program, Harvard school of Public Health, 677 Huntington Avenue, Boston, MA 02115, USA; 4 Pulmonary and Critical Care Unit, Massachusetts General Hospital, Bulfinch 148, 55 Fruit Street, Boston, MA 02114, USA; 5 Department of Health Services Administration, China Medical University, No. 91, Hsueh-Shih Road, Taichung 404, Taiwan; 6 Athletics Department & Graduate School, National Taiwan College of Physical Education, No. 16, Sec. 1, Shuang-Shih Road, Taichung 404, Taiwan; 7 School of Public Health, China Medical University, No. 91, Hsueh-Shih Road, Taichung 404, Taiwan; 8 China Medical University Beigang Hospital, No. 123, Shin-Der Road, Beigang Town, Yunlin County 651, Taiwan; 9 Institute of Population Health Sciences, National Health Research Institutes, No. 35, Keyan Road, Zhunan Town, Miaoli County 350, Taiwan; 10 Institute of Hospital and Health Care Administration, School of Medicine, National Yang-Ming University, No.155, Sec.2, Linong Street, Taipei 112, Taiwan

**Keywords:** General health, Qigong, quality of life, Waitankung

## Abstract

**Background:**

Qigong, similar to Tai Chi Chuan, is beneficial to health. In Taiwan, Waitankung, a type of Qigong, is as popular as Tai Chi Chuan. This population-based comparison study compares the health-related quality of life between people practicing Waitankung and their comparable community residents.

**Methods:**

A total of 165 individuals practicing Waitankung were matched by age and sex with 660 general individuals for comparison. Information about health-related quality of life, measured by the SF-36, and other basic and health conditions was obtained from the questionnaires. This study used the linear mixed-effect regression model to examine the association between health-related quality of life and the practice of Waitankung.

**Results:**

Compared with either sedentary individuals or individuals practicing other types of exercise, the Waitankung group scored higher for eight and five out of ten SF-36 components, respectively. The Waitankung group scored better in general health, vitality, and physical component summary compared to individuals participating in other types of exercise, even when considering the energy expended by exercise.

**Conclusion:**

The results suggest that Waitankung exercising is significantly associated with health-related quality of life. Waitankung may serve as an exercise choice for middle-aged and older people to improve overall quality of life.

## Background

Among the tools for promoting health, physical activity plays an essential role because of its beneficial effect on health, including reduced risk of mortality and developing cancer, type II diabetes, and high blood pressure in the general population, and geriatric conditions in the older population [1]. In Chinese society, Tai Chi Chuan and Qigong are two popular physical activities, mainly because of their potential physical and mental benefits and gentle movements suitable for middle-aged and old people. Several studies have found that Tai Chi Chuan and Qigong provide health benefits similar to other physical activities [2,3]. For example, in a recent review of randomized control trials for Tai Chi Chuan and Qigong, both provide similar health benefits of bone density, cardiopulmonary effects, physical function, falls, quality of life, self-efficacy, reported health outcomes, psychological symptoms, and immune function [3].

**Figure 1:**
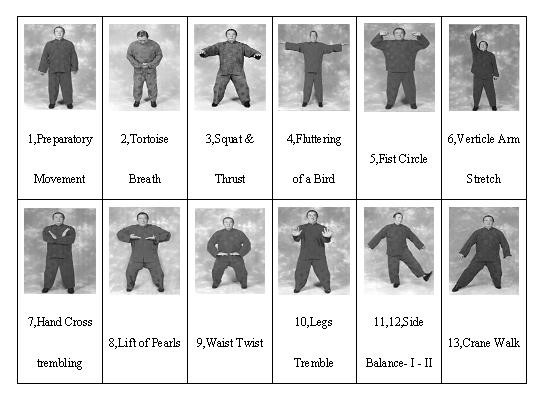
**Typical WTK twelve movements**. (Demonstrated by CT Chang, WTK ancestor master).

In Taiwan, Waitankung (WTK), a form of Qigong, is as popular as Tai Chi Chuan. WTK has one preliminary movement and eleven movements (Figure 1). The main purpose of these movements is to activate the 'qi' inside the body, flow it through all parts of the body, and make the body tremble to yield a qi field in the body [4]. Regular practice of these movements enhances the body to a stable condition, improving different organ-systems including the nervous, musculoskeletal, and circulatory systems as well as the mental and cognitive status [4].

Research has not adequately investigated the underlying mechanism of WTK on health. Based on the measurement of heart rate variability and hemodynamics, results of a recent study indicated that WTK, as compared to inactivity or other physical activities, enhances vagal modulation and suppresses sympathetic modulation after 30 minutes of practice, and enhances sympathetic modulation without compromising vagal modulation after 60 minutes of practice [5].

Both Tai Chi Chuan and Qigong improve quality of life. However, no study has investigated overall health effects, as measured by health-related quality of life, on people who regularly practice WTK. Whether WTK provides similar benefits remains unclear. Thus, this work conducts a population-based study comparing the health-related quality of life in people practicing WTK with the general population in Taiwan.

## Methods

### Study and comparison groups

WTK is a physical activity commonly practiced in Taiwan. This study obtained a list of all members joining the WTK association. Those who had practiced for over half a year or regularly practiced at least two hours per week for 26 weeks, and were 40 and over at the time of study were eligible for enrolment. Based on the inclusion criteria, the current research chose 194 individuals of all eligible members from the list as the study group. Of them, 29 did not agree to participate in the study or complete the questionnaire, leaving 165 individuals in the study. The questionnaire designed for the study group consisted of socio-demographic information, health-related quality of life, lifestyle behaviours including WTK and other physical activities, and medical history. Individuals from participants of the National Health Interview Survey (NHIS) conducted in 2001 were chosen to compare with the study group. The NHIS is a nationally representative study of general health in Taiwan. The 2001 NHIS included 5798 households and 18143 study participants aged 12 and over [6]. For each WTK practitioner, four comparison subjects were matched for age (within five years) and gender. Thus, 660 participants of the NHIS were selected as the comparison group. The study was reviewed and approved by the Institutional Review Board of the principal investigator institution. All individuals in the study group were fully informed through regular meetings of WTK members and agreed to participate in the study. The NHIS data used for comparison has been released for public use and can be obtained through http://nhis.nhri.org.tw/.

### Measurement

Health-related quality of life was assessed using the Taiwanese version of Short Form 36 (SF-36) for both study and comparison groups [7]. The Taiwanese version has been tested with validity results similar to those in the US and other language versions [8,9]. The SF-36 measures eight domains of health including physical functioning, role limitations due to physical problems, bodily pain, general health, vitality, social functioning, role limitations due to emotional problems, and general mental health [7]. Items in each domain were aggregated and transformed into a 0 to 100 scale, with higher values representing better health status. In addition to the score for each domain, two summary scores were also created [10]. The physical component summary was formed by physical functioning, role limitations due to physical problems, bodily pain, and general health, while the mental component summary was formed by general mental health, role limitations due to emotional problems, social functioning, and vitality.

Other personal health-related information included in the reported questionnaire was education, alcohol use, smoking, leisure-time physical activity, and chronic diseases covering stroke, diabetes, heart disease, and hypertension. Education was dichotomized as high school or less and college or above. Alcohol use was categorized based on the frequency of current alcohol consumption as rarely, occasionally, and regularly. Cigarette smoking was divided into three groups as never, ever, and current smoking. Leisure-time physical activity was first classified as exercisers or sedentary individuals. Exercisers were further calculated according to total energy expended from all leisure-time physical activities engaged in a week. Energy expenditure value for each activity including WTK was assigned according to activity intensity (Metabolic Equivalent Task; MET) assessed in Taiwan [11]. The MET for each activity where the exerciser had a slight increase in breathing was chosen. For example, the METs for WTK and leisure walking, both with a slight increase in breathing, were 3.8 and 3.3, respectively. Energy expenditure of each activity per week was calculated by MET (kcal/min) × frequency (times) × duration for each time (minutes), assuming 60 kg of weight for each individual. Energy expenditure of each activity was added up into total amount of energy expenditure. For instance, an individual engaged in 30 minutes of leisure walking seven times a week and one hour of WTK four times a week, expended a total weekly energy of: (3.3 × 7 × 30) + (3.8 × 4 × 60) = 693 + 912 = 1605 kcal. The total amount of energy expenditure in calories was further divided into three groups with no, less than 1000 kilocalories per week, and equal or greater than 1000 kilocalories per week, which was roughly tertiled for each category. Disease history consisted of with or without a history of stroke, diabetes, heart disease, and hypertension. The questionnaire also asked the study group about the cumulative years of practicing WTK.

### Statistical analysis

Descriptive statistics of baseline characteristics for both study and comparison groups were computed with percents for categorical variables (gender, age, education, alcohol use, smoking, exercise, four selected diseases, and practice year) and the means ± standard deviations for continuous variables (ten components of SF-36 and practice year). Statistical differences between the two groups were also analyzed using the χ^2 ^test for categorical variables and the t-test for continuous variables, with Bonferroni post-test correction. The current work used linear mixed-effect regression models to determine whether WTK practice was associated with increased scores in the ten components (eight domains and two summaries) of SF-36. The basic model categorized people in the study and comparison groups as no exercise, WTK exercise, and other types of exercise, according to their exercise status. Other covariates adjusted in the model consisted of three demographic factors (gender, age, and education), two lifestyle factors (alcohol consumption and cigarette smoking), and four chronic diseases (stroke, diabetes, heart disease, and hypertension). To explore the relationship between WTK and health-related quality of life in more detail, two subsequent analyses were conducted separately for all exercisers, in which calorie expenditure was considered in the model, and for the study group, in which practicing year was added to examine the cumulative effect of WTK. All statistical analyses were performed with SAS. P values for all tests were two-tailed, and statistical differences were considered at the < 0.05 level.

## Results

Table 1 presents the descriptive details of the studied sample. The age of study participants ranged from 44 to 90. A higher percentage of females was present than for males in both groups. The three demographic factors (gender, age, and education) did not differ between the two groups, suggesting that both groups are comparable. People practicing WTK appeared to have healthier lifestyle behaviours including adequate alcohol intake, no cigarette smoking, and performing exercise. Not surprisingly, the prevalence of chronic diseases including diabetes, heart disease, and hypertension was significantly lower in the study group, compared to that in the comparison group. Similarly, the scores of seven out of ten components in the SF-36, except for those in the domains of social functioning, role limitations due to emotional problems, and mental component summary, were significantly higher in the study group.

**Table 1:** Characteristics of study and comparison groups

**Variables/n (%) or mean ± S.D**.	Study group (n = 165)	Comparison group (n = 660)	*p *value
Gender			
Male	68(41.2)	272(41.2)	1.000
Female	97(58.8)	388(58.8)	
Age			
< 60	67(40.6)	223(33.8)	0.200
60-69	55(33.3)	227(34.4)	
≥ 70	43(26.1)	210(31.8)	
Education			
High school or less	117(70.9)	449(68.0)	0.476
College or above	48(29.1)	211(32.0)	
Alcohol use			
Rarely	128(77.6)	547(82.9)	0.001
Occasionally	31(18.8)	62(9.4)	
Regularly	6(3.6)	51(7.7)	
Smoking			
Never	131(79.4)	515(78.0)	0.018
Ever	20(12.1)	47(7.1)	
Current	14(8.5)	98(14.9)	
Exercise status			
No	0(0.0)	206(31.2)	< .001
Yes	165(100.0)	454(68.8)	
Exercise by calories			
No exercise	0(0.0)	206(31.2)	< .001
< 1000 kcal/week	26(15.8)	257(38.9)	
≥ 1000 kcal/week	139(84.2)	197(29.9)	
Stroke			
No	164(99.4)	642(97.3)	0.146
Yes	1(0.6)	18(2.7)	
Diabetes			
No	156(94.6)	580(87.9)	0.014
Yes	9(5.5)	80(12.1)	
Heart disease			
No	159(96.4)	538(81.5)	< .001
Yes	6(3.6)	122(18.5)	
Hypertension			
No	138(83.6)	441(66.8)	< .001
Yes	27(16.4)	219(33.2)	
Practice year	12.4 ± 7.9		
< 10 years	64(38.8)		
10~20 years	76(46.1)		
> 20 years	25(15.2)		
SF-36 scale			
PF	86.8 ± 14.9	78.6 ± 23.5	< .001
RP	82.3 ± 31.5	65.8 ± 43.5	< .001
BP	82.3 ± 15.1	74.9 ± 22.9	< .001
GH	75.8 ± 17.4	59.3 ± 22.7	< .001
VT	72.1 ± 16.7	62.8 ± 20.1	< .001
SF	84.1 ± 14.2	82.4 ± 20.9	0.211
RE	79.2 ± 33.8	75.4 ± 39.5	0.215
MH	76.5 ± 14.6	72.3 ± 17.4	0.002
PC	52.0 ± 5.9	46.3 ± 10.4	< .001
MC	50.9 ± 7.8	49.9 ± 9.6	0.154

Notes:

1. PF, physical functioning; RP, role limitations due to physical problems; BP, bodily pain; GH, general health; VT, vitality; SF, social functioning; RE, role limitations due to emotional problems; MH, general mental health; PC, physical component summary; MC, mental component summary.

2. p values were calculated with chi-square test for categorical variables, and t-test with Bonferroni post-test correction for continuous variables.

Table 2 shows the health-related quality of life of people practicing WTK as related to the quality of life of the comparable general population. To better compare exercise status with the study group, persons in the comparison group were divided into no exercise and exercise with types other than WTK. After adjusting for the effects of potential confounders, significantly higher scores were observed for persons practicing WTK in eight components (except social functioning and role limitations due to emotional problems) compared to sedentary people. WTK people had significantly better scores in five components (except physical functioning, social functioning, role limitations due to emotional problems, general mental health, and mental component summary) as compared to those participating in other types of exercise.

**Table 2:** Multiple mixed-effect regression model assessing the adjusted association between exercise status and scores of SF-36 (n = 825)

Variable/Coefficient (standard deviation)	PF	RP	BP	GH	VT	SF	RE	MH	PC	MC
Exercise status										
No										
Yes, other types	6.5(1.6)***	10.6(3.3)**	4.4(1.7)*	6.1(1.7)***	7.8(1.6)***	4.2(1.6)**	9.1(3.2)**	4.9(1.4)***	2.6(0.7)***	2.7(0.8)***
Yes, WTK	9.2(2.0)***	18.5(4.1)***	8.3(2.2)***	17.1(2.1)***	13.0(1.9)***	3.1(2.0)	6.0(4.0)	6.3(1.7)***	5.9(0.9)***	2.2(1.0)*
Yes, other types^a^										
No^a^	-6.5(1.6)***	-10.6(3.3)**	-4.4(1.7)*	-6.1(1.7)***	-7. 8(1.6)***	-4.2(1.6)**	-9.1(3.2)**	-4.9(1.4)***	-2.6(0.7)***	-2.7(0.8)***
Yes, WTK^a^	2.8(1.8)	7.9(3.6)*	3.8(1.9)*	11.0(1.8)***	5.2(1.7)**	-1.2(1.8)	-3.1(3.5)	1.4(1.51)	3.3(0.8)***	-0.4(0.9)

Notes:

1. ^a^Other types of exercise was set as the reference group in the models.

2. * P < 0.05; ** P < 0.01; *** P < 0.001

3.PF, physical functioning; RP, role limitations due to physical problems; BP, bodily pain; GH, general health; VT, vitality; SF, social functioning; RE, role limitations due to emotional problems; MH, general mental health; PC, physical component summary; MC, mental component summary; WTK, Waitankung

4. Other variables controlled in the model in addition to the intercept were gender, age, education, alcohol use, smoking, stroke, diabetes, heart disease, and hypertension.

This work further limited the analysis to compare health-related quality of life among those practicing WTK versus those practicing other types of exercise only. After considering the effect of energy expended from exercise, results in Table 3 indicate that the significantly higher scores for people with WTK remained evident for the domains of general health, vitality, and physical component summary, but not for the other two domains (role limitations due to physical problems and bodily pain).

**Table 3:** Multiple mixed-effect regression model assessing the adjusted association between exercise status and scores of SF-36 in exercisers (n = 619)

Variables/Coefficient (standard deviation)	PF	RP	BP	GH	VT	SF	RE	MH	PC	MC
Exercise status										
Yes, other types										
Yes, WTK	1.4 (1.8)	3.0 (3.7)	2.5 (1.9)	9.6 (1.9) ***	4.4 (1.8)*	-2.3 (1.8)	-3.7 (3.6)	1.3 (1.6)	2.2 (0.8)**	-0.4 (0.9)
Exercise by calories										
< 1000 kcal/week										
≥ 1000 kcal/week	3.58 (1.5)*	10.4 (3.1)**	3.5 (1.6)*	2.5 (1.7)	1.6 (1.5)	2.3 (1.5)	0.69 (3.0)	0.0 (1.4)	2.5 (0.7) ***	-0.3 (0.8)

Notes:

1. * P < 0.05; ** P < 0.01; *** P < 0.001

2.PF, physical functioning; RP, role limitations due to physical problems; BP, bodily pain; GH, general health; VT, vitality; SF, social functioning; RE, role limitations due to emotional problems; MH, general mental health; PC, physical component summary; MC, mental component summary; WTK, Waitankung

3. Other variables controlled in the model in addition to the intercept were gender, age, education, alcohol use, smoking, stroke, diabetes, heart disease, and hypertension.

Finally, to test cumulative effect for the study group on health-related quality of life, the practicing year was further analyzed in the model (Table 4). The results revealed that only general mental health scored slightly higher in people practicing 10 to 20 years, compared to people in other groups, indicating that the length of practicing did not determine improved health-related quality of life from WTK.

**Table 4:** Multiple mixed-effect regression model assessing the adjusted association between practicing years and scores of SF-36 in the study group (n = 165)

Variables/Coefficient (standard deviation)	PF	RP	BP	GH	VT	SF	RE	MH	PC	MC
Practice year										
< 10 years										
10~20 years	-3.2 (2.8)	-10.1 (5.8)	-0.5 (2.9)	2.8 (3.34)	2.6 (3.2)	-1.1 (2.7)	-7.9 (6.3)	6.0 (2.8)*	-1.6 (1.1)	1.4 (1.5)
> 20 years	3.5 (3.7)	1.4 (7.8)	1.8 (3.9)	-1.7 (4.5)	-0.7 (4.3)	-5.1 (3.6)	-8.7 (8.5)	3.01 (3.7)	1.0 (1.5)	-1.5 (2.0)

Notes:

1. * P < 0.05; ** P < 0.01; *** P < 0.001

2.PF, physical functioning; RP, role limitations due to physical problems; BP, bodily pain; GH, general health; VT, vitality; SF, social functioning; RE, role limitations due to emotional problems; MH, general mental health; PC, physical component summary; MC, mental component summary; WTK, Waitankung

3. Other variables controlled in the model in addition to the intercept were gender, age, education, alcohol use, smoking, stroke, diabetes, heart disease, and hypertension.

## Discussion

In this community-based comparison study of middle-aged and older Taiwanese adults, practicing WTK was significantly associated with health-related quality of life. People practicing WTK had better ratings in many components of SF-36, particularly the components of general health, vitality, and physical component summary, compared to sedentary individuals or even non-WTK exercisers. The data further revealed that the length of practicing WTK did not associate with further improvement of health-related quality of life.

In contrast to the large number of publications on Tai Chi Chuan and health benefits, relatively little research has been conducted on Qigong or WTK and health. Results from recent studies of Tai Chi Chuan and quality of life have indicated that Tai Chi Chuan relates to improvements in certain components of quality of life, as measured by SF-36 [12-15]. Although no such study has been undertaken for WTK, we found that WTK improves quality of life similar to Tai Chi Chuan. Given that both Tai Chi Chuan and WTK are peaceful, slow, and graceful movements suitable for the elderly, our results are not surprising. Further longitudinal studies are needed to investigate the actual mechanisms of WTK to improve overall health, and to further compare the health benefits of WTK with Tai Chi Chuan.

Although people practicing WTK had better quality of life compared to those who were sedentary or with other types of physical activity, the years practicing WTK were not associated with quality of life among WTK practitioners. The data suggest that practicing WTK provides consistent health effect so that both short-and long-term practitioners receive similar overall health benefits. Nonetheless, caution needs to be exercised when interpreting this result. The long-term effect of practicing WTK on health is possibly not on quality of life measured by SF-36, but on other domains of physiological health. The observed indifference could also be influenced by the small sample of WTK participants.

This study is the first, to our knowledge, to examine the association between practicing WTK and improved health-related quality of life. The data were based on a validated instrument measuring health-related quality of life. The model adjusted for a number of factors that were potentially confounding for the association, and considered the possible effect of exercise and its energy expenditure. However, the weak points should be noted. Firstly, measurement errors potentially existing in the information obtained from self-reported questions including quality of life and health behaviours, such as exercise and alcohol use included in our study, might have biased the association between WTK and quality of life. However, the bias may be non-differential between the study and comparison groups and would tend to result in underestimation of the differences in the SF-36 scores between the two groups. Secondly, persons practicing WTK were members of the WTK association only and the study sample may not be representative of all people practicing WTK. Thirdly, because the health-related information obtained for the study and comparison groups was from two different data, it is possible that reporting bias exists in our study and may lead to under-reporting unexpected results for WTK practitioners. Fourthly, certain health-related factors that could also affect health-related quality of life, such as reported health, Parkinson's disease, and lung disease were not considered. The exclusion of these factors could inevitably yield potential bias in comparing the quality of life between the two groups. Finally, data analyzed in this study came from a cross-sectional survey and, as a result, it is not able to make causal inferences from our associations.

## Conclusions

In conclusion, this cross-sectional study represents the first report from a community-based comparison study of WTK and quality of life. WTK practitioners have better health-related quality of life than their counterparts have. Because older people are limited by aging-related functional decline, choosing a physical activity that is suitable and has the benefit of overall health is important for the elderly. In this regard, WTK is worthy of considering as an exercise choice for middle-aged and elderly populations.

## Competing interests

The authors declare that they have no competing interests.

The study was supported by the China Medical University (Grant No. CMU94-025).

## Authors' contributions

TJH, JSL, and TYL, participated in designing the study, collected and analysed data, wrote the first draft of the paper, and led the critical review and revision of the paper. CHL and YCY contributed to the design of study, collected and analysed data, and contributed to the drafting and critical reviewing of the paper. The remaining authors (DCC, TCM, TRJ, SZL, and JGL.) made substantial contributions to the conception and design, interpretation of data, and critically revised the intellectual content of the manuscript. All authors have approved the final version of the manuscript.

## Pre-publication history

The pre-publication history for this paper can be accessed here:

http://www.biomedcentral.com/1471-2458/11/546/prepub
